# ACCI could be a poor prognostic indicator for the in-hospital mortality of patients with SFTS

**DOI:** 10.1017/S0950268823001930

**Published:** 2023-12-06

**Authors:** Chen Gong, Xinjian Xiang, Baoyu Hong, Tingting Shen, Meng Zhang, Shichun Shen, Shenggang Ding

**Affiliations:** 1 Department of Pediatrics, The First Affiliated Hospital of Anhui Medical University, Hefei, China; 2 Department of Cardiology, The First Affiliated Hospital of USTC, Division of Life Sciences and Medicine, University of Science and Technology of China, Hefei, China; 3 Department of Plastic and Reconstructive Surgery, The Second Affiliated Hospital of Anhui Medical University, Hefei, China; 4 Department of Pediatrics, The Second Affiliated Hospital of Anhui Medical University, Hefei, China; 5 Department of Pathology, the First Affiliated Hospital of Anhui Medical University, Hefei, China; 6 Department of Cardiology, The Second Affiliated Hospital of Anhui Medical University, Hefei, China

**Keywords:** SFTS, ACCI, in-hospital mortality, *Bunyaviridae*, infectious disease

## Abstract

This study aims to evaluate the predictive role of age-adjusted Charlson comorbidity index (ACCI) scores for in-hospital prognosis of severe fever in thrombocytopenia syndrome (SFTS) patients. A total of 192 patients diagnosed with SFTS were selected as the study subjects. Clinical data were retrospectively collected. Receiver operating characteristic curves were used to evaluate the diagnostic value of ACCI for the mortality of SFTS patients, and Cox regression models were used to assess the association between predictive factors and prognosis. The 192 SFTS patients were divided into two groups according to the clinical endpoints (survivors/non-survivors). The results showed that the mortality of the 192 hospitalized SFTS patients was 26.6%. The ACCI score of the survivor group was significantly lower than that of the non-survivor group. Multivariate Cox regression analysis showed that the increased ACCI score was a significant predictor of poor prognosis in SFTS. Kaplan–Meier survival analysis showed that SFTS patients with an ACCI >2.5 had shorter mean survival times, indicating a poor prognosis. Our findings suggest that ACCI, as an easy-to-use clinical indicator, may offer a simple and feasible approach for clinicians to determine the severity of SFTS.

## Introduction

Severe fever with thrombocytopenia syndrome (SFTS) is an infectious zoonotic disease caused by the SFTS virus (SFTSV), a recently discovered member of the Phlebovirus genus in the Bunyaviridae family. SFTSV is divided into six genotypes (A-F) and was first isolated from a patient in Hubei, China, in 2009. Subsequently, the same virus was identified in multiple patients and was confirmed to be SFTSV [1–3]. SFTS has mainly been reported in countries such as China, South Korea, Japan, and Pakistan [4–6], with major symptoms including fever, leukopenia, thrombocytopenia, and gastrointestinal symptoms. This disease is highly contagious, with a mortality of up to 30% [2, 7]. The primary transmission route of SFTS is believed to be through tick bites [1, 8]. Nevertheless, transmission through contact with infected animals and patients, as well as sexual transmission between humans, has also been reported [9–11].

Early identification of SFTS in patients with a high risk of death can help implement efficient clinical approaches, thereby reducing disease severity and mortality. Comorbidities affect the mortality rate of patients with infectious diseases and may also influence their treatment plans. Previous studies have shown that information on the patient’s health status and the number of comorbidities involved can help determine the prognosis of the disease [12]. Therefore, a comprehensive indicator of the patient’s basic health status may be useful. One standardized method for assessing comorbidities is the Charlson comorbidity index (CCI), which was first proposed in 1987 and developed based on 17 potential conditions to predict one-year and ten-year all-cause mortality [13].

CCI is the most widely used comorbidity index and has been validated in many clinical settings [14, 15]. The clinical measurement sensitivity of CCI has also been proven under various medical conditions, and increases in CCI scores correlate with an increase in mortality. Furthermore, CCI has incremental clinical measurement properties, and adding CCI to other measurements can improve the overall predictive accuracy [16]. Studies have shown that CCI can predict long-term mortality in different clinical populations, including internal medicine, surgery, and intensive care units (ICUs) [16]. It is also a reliable predictor of in-hospital mortality for infectious diseases such as acute respiratory infections and community-acquired pneumonia [12, 17].

However, no studies have yet focussed on the predictive effect of comorbidities on adverse outcomes in hospitalized SFTS patients. In addition, studies have shown that adverse outcomes in SFTS patients are significantly associated with the patient’s age. Zhang et al.’s study showed that the risk of death in SFTS patients over 65 years old increased by 3.384 times [18]. Due to advances in treatment and disease management, CCI has undergone various refinements and has been validated in different studies [19–21]. Among them, the age-adjusted Charlson comorbidity index (ACCI) score, which incorporates age as an additional indicator, has also been proven to be a good predictor of adverse outcomes in COVID-19 patients [22]. Therefore, the aim of this study is to identify predictors of outcomes in hospitalized SFTS patients and to explore the effectiveness of ACCI as a prognostic indicator.

## Materials and methods

### Study design and patient selection

This study was performed in the First Affiliated Hospital of Anhui Medical University. One hundred ninety-two patients with SFTS who were hospitalized between April 2020 and January 2023 were enrolled in this study. Patient demographic and clinical data were collected from the electronic medical record system. This study complied with all relevant national regulations and was approved by the Ethics Committee of the First Affiliated Hospital of Anhui Medical University (Ethics Approval No. PJ 2023-2107-50). In this retrospective study, informed consent was waived by using anonymous clinical data. The confidentiality of all participants’ personal information was safeguarded.

### Diagnostic criteria

According to the criteria published by the Chinese Ministry of Health in 2010, SFTS is defined as patients with fever ≥38.0 °C, bleeding and/or gastrointestinal symptoms, epidemiological risk factors (including exposure to ticks and/or contact with SFTS endemic areas), and laboratory results including thrombocytopenia. The diagnosis is confirmed by positive viral nucleic acid detection by RT-PCR or positive SFTSV-specific IgM/IgG antibodies [23, 24]. The exclusion criteria were patients who died within 24 h of admission, those aged below 16 years, and those with incomplete clinical data.

### Laboratory test

Data on demographic characteristics and laboratory findings of all SFTS patients upon admission were collected through the electronic medical record system, including routine blood parameters, biochemical indicators, and coagulation function indicators. Blood samples for laboratory tests were collected within 24 h after the patients were admitted (using EDTA tubes, sodium citrate tubes, or coagulation tubes). Blood collected with EDTA was immediately tested for routine blood parameters. Blood collected with sodium citrate underwent coagulation function tests after being centrifuged at 3500 r/s for 15 min, and blood without anticoagulant underwent biochemical tests after being centrifuged at 3500 r/s for 5 min.

### ACCI score evaluation

In this study, SFTS patients were classified according to the original Charlson index [13] for comorbidities. The Charlson index considers 17 different comorbidities, with scores ranging from 1 to 6 points for each comorbidity, and the final CCI score for each patient is the sum of all comorbidity scores. The CCI score was calculated based on the patient’s medical history at admission, and diagnoses made after admission were not included in the scoring. The ACCI score was then formed by incorporating age as an additional indicator in the modified version from 1994 [19]. Detailed definitions and calculation methods of CCI are provided in the Supplementary Material.

### Statistical analysis

The normality of data distribution for continuous variables was assessed using a Kolmogorov–Smirnov test. Normally distributed variables were expressed as mean ± standard deviation (SD), while skewed variables were expressed as median (25th–75th percentiles). Between-group data comparisons were conducted through either a t-test or a non-parametric Mann–Whitney U test. The chi-square test or Fisher’s exact probability test was used to compare differences for categorical variables.

Multivariate Cox regression analysis was used to identify factors associated with the in-hospital mortality of SFTS. The entry time was the time of admission, and the exit time was the time of either in-hospital death or discharge. The event was defined as death during hospitalization. No violations of proportional hazard assumption were found (*p* > 0.99). Using a stepwise regression approach, variables with a *p* < 0.1 in the univariate Cox regression analysis were included in the multivariate Cox regression to determine the independent factors affecting in-hospital mortality in SFTS patients.

Receiver operating characteristic (ROC) curves were plotted, and the area under the curve (AUC) was used to evaluate the predictive ability of the four Charlson comorbidity index scores. Based on the optimal cutoff value of the ACCI score in the ROC analysis, patients were stratified into high- and low-risk groups. Kaplan–Meier survival curves were plotted, and the log-rank test was used to analyze the survival differences between the two groups.

SPSS 25.0 software (SPSS Inc., Chicago, IL, USA), GraphPad Prism (version 9.0) and R (version 4.1.2) were used for statistical analyses and graphic depiction. The values of *p* < 0.05 were considered statistically significant.

## Results

### Comparison of demographics and clinical characteristics between survivors and non-survivors

This study included 192 hospitalized SFTS-confirmed patients from April 2020 to January 2023, including 84 males (43.7%) and 108 females (56.3%). The median age of patients was 64.00 (54.25, 71.00) years. The case fatality rate was 26.6% (51/192), with multiple organ dysfunction being the main cause of death.

The comparison of clinical characteristics between SFTS survivors and non-survivors was shown in Table 1. Survivors had a median age of 61 years, compared to 71 years for non-survivors; a statistically significant difference was found (*p* < 0.001). The most common symptoms of SFTS patients included fever, rash, digestive system symptoms, respiratory system symptoms, and central nervous system symptoms. Among them, there was a significant difference in neurological symptoms between the two groups, with a higher proportion of central nervous system symptoms in non-survivors (33.3%) than in survivors (14.9%) (*p* = 0.007). In addition, there was no significant difference in smoking and drinking history between the two groups. Nevertheless, the proportion of hypertensive patients in the non-survivor group was significantly higher than that in the survivor group (*p* = 0.015).

**Table 1.** Participant clinical characteristics between survivors and non-survivors of SFTS

Characteristics	Survivors (n = 141)	Non-survivors (n = 51)	*p* value
Age (years)	61 (53, 68)	71 (64, 75)	<0.001
Male, n (%)	64 (45.4%)	20 (39.2%)	0.511
Time from onset to admission (days)	5 (3, 7)	4 (3, 7)	0.329
Clinical symptoms, n (%)			
Respiratory symptoms	17 (12.1%)	4 (7.8%)	0.452
Gastrointestinal symptoms	83 (58.9%)	27 (52.9%)	0.511
CNS symptoms	21 (14.9%)	17 (33.3%)	0.007
Fever	134 (95.0%)	49 (96.0%)	1.000
Rash	92 (65.2%)	29 (56.9%)	0.313
Past history, n (%)			
Hypertension	28 (19.9%)	19 (37.3%)	0.015
Drinking	14 (10.0%)	6 (11.8%)	0.790
Smoking	10 (7.1%)	7 (13.7%)	0.248
Haematological parameters			
WBC (× 10^9^/L)	2.72 (1.68, 4.68)	2.32 (1.37, 3.40)	0.026
N (× 10^9^/L)	1.53 (0.95, 2.89)	1.47 (0.80, 2.13)	0.193
L (× 10^9^/L)	0.71 (0.47, 1.19)	0.51 (0.40, 0.74)	0.002
M (× 10^9^/L)	0.15 (0.08, 0.32)	0.09 (0.06, 0.17)	0.001
PLT (× 10^9^/L)	55.00 (39.50, 78.00)	38.00 (27.00, 50.00)	<0.001
HB (g/L)	130.83 ± 18.81	128.92 ± 19.20	0.538
ALP (U/L)	68.00 (56.00, 86.50)	73.00 (56.00, 101.00)	0.307
LDH (U/L)	770.00 (489.00, 1,212.00)	1,545.00 (868.00, 2,929.00)	<0.001
ALT (U/L)	69.00 (43.50, 98.50)	113.00 (63.00, 181.00)	0.001
TBIL (μmol/L)	10.70 (8.40,13.25)	10.00 (8.50, 14.20)	0.731
Glu (mmol/L)	6.60 (5.59, 8.84)	7.89 (6.00, 13.00)	0.012
Alb (g/L)	34.46 ± 5.20	31.96 ± 4.15	0.002
CK-MB (U/L)	19.00 (10.50, 33.00)	23.00 (12.00, 54.00)	0.036
eGFR (ml/(min·1.73 m^2^))	96.00 (83.00, 106.00)	65.00 (47.00, 89.00)	<0.001
K (mmol/L)	3.68 ± 0.54	3.92 ± 0.55	0.009
Na (mmol/L)	135.00 (132.20, 137.70)	133.60 (132.10, 136.60)	0.123
Ca (mmol/L)	1.98 (1.90, 2.07)	1.88 (1.79, 1.99)	<0.001
Mg (mmol/L)	0.85 ± 0.10	0.84 ± 0.10	0.613
HCO3- (mmol/L)	24.63 ± 3.53	22.40 ± 4.32	<0.001
aPTT (s)	47.50 (41.35, 54.85)	59.70 (52.00, 75.90)	<0.001
PT (s)	13.00 (12.50, 13.60)	13.60 (12.90, 14.80)	<0.001
FIB (g/L)	2.69 (2.25, 3.12)	2.34 (2.05, 2.70)	0.002
FDP (μg/mL)	5.72 (3.25, 11.50)	27.08 (12.46, 40.59)	<0.001

*Note:* Continuous variable data are presented as the mean (± SD) or the median (*P_25_, P_75_*). Classified variable data are presented as n (%). *p* values are based on comparisons between the group of survivors and the group of non-survivors.

Abbreviations: Alb, albumin; ALP, alkaline phosphatase; ALT, alanine aminotransaminase; aPTT, activated partial thromboplastin time; CK-MB, creatine phosphokinase isoenzyme; CNS, central nervous system; eGFR, estimated glomerular filtration rate; FDP, fibrinogen degradation product; Glu, glucose; HB, Haemoglobin; L, lymphocyte; LDH, lactate dehydrogenase; M, monocyte; N, neutrophil; PLT, platelet; PT, prothrombin time; SFTS, severe fever with thrombocytopenia syndrome; TBIL, total bilirubin; WBC, white blood cell.

Statistical analysis of the laboratory examination results of SFTS patients showed that, compared with survivors, non-survivors had lower white blood cell counts, lymphocyte counts, PLT counts, Alb, eGFR, blood calcium, bicarbonate, and FIB and higher levels of LDH, ALT, Glu, CK-MB, blood potassium, aPTT, PT, and FDP (Table 1).

### Comparison of ACCI between survivors and non-survivors of SFTS

Table 2 summarizes the contributions of each factor to the final ACCI scores for the two groups. Among the 192 SFTS patients, 72 patients (37.5%) had at least one complication. The most common comorbidities were diabetes mellitus in 44 patients (22.9%), followed by cerebrovascular disease (11.5%), mild liver disease (5.2%), and peripheral vascular disease (3.1%). Compared with SFTS survivors, the proportions of cerebrovascular disease (31.4%) were significantly higher among non-survivors (Table 2).

**Table 2.** Comparison of ACCI with score weights between survivors and non-survivors of SFTS

Comorbidity	Score	Total (n = 192)	Survivors (n = 141)	Non-survivors (n = 51)	*p* value
Myocardial infarction	1	2 (1.0%)	0 (0.0%)	2 (3.9%)	0.070
Chronic cardiac failure	1	2 (1.0%)	0 (0.0%)	2 (3.9%)	0.070
Peripheral vascular disease	1	6 (3.1%)	1 (0.7%)	5 (9.8%)	0.005
Cerebrovascular disease	1	22 (11.5%)	6 (4.3%)	16 (31.4%)	<0.001
Dementia	1	0 (0.0%)	0 (0.0%)	0 (0.0%)	–
Chronic pulmonary disease	1	2 (1.0%)	1 (0.7%)	1 (2.0%)	0.046
Connective tissue disease	1	3 (1.6%)	1 (0.7%)	2 (3.9%)	0.173
Peptic ulcer	1	5 (2.6%)	4 (2.8%)	1 (2.0%)	1.000
Mild liver disease	1	10 (5.2%)	7 (5.0%)	3 (5.9%)	1.000
Diabetes mellitus	1	44 (22.9%)	28 (19.9%)	16 (31.4%)	0.119
Hemiplegia	2	1 (0.5%)	0 (0.0%)	1 (2.0%)	0.266
Diabetes with end-organ damage	2	0 (0.0%)	0 (0.0%)	0 (0.0%)	–
Moderate/severe renal disease	2	0 (0.0%)	0 (0.0%)	0 (0.0%)	–
Tumour/Leukaemia/Lymphoma	2	0 (0.0%)	0 (0.0%)	0 (0.0%)	–
Moderate/severe liver disease	2	0 (0.0%)	0 (0.0%)	0 (0.0%)	–
Metastatic solid tumour/AIDS	6	0 (0.0%)	0 (0.0%)	0 (0.0%)	–
Age					<0.001
<50 years	0	28 (14.6%)	27 (19.1%)	1 (2.0%)	
50–59 years	1	41 (21.4%)	38 (27.0%)	3 (5.9%)	
60–69 years	2	69 (35.9%)	52 (36.9%)	17 (33.3%)	
70–79 years	3	49 (25.5%)	24 (17.0%)	25 (49.0%)	
≥80 years	4	5 (2.6%)	0 (0.0%)	5 (9.8%)	

Abbreviations: ACCI, age-adjusted Charlson Comorbidity Index; AIDS, acquired immune deficiency syndrome.

### Distinct Charlson comorbidity scores for survivors and non-survivors of SFTS

**Figure 1.**
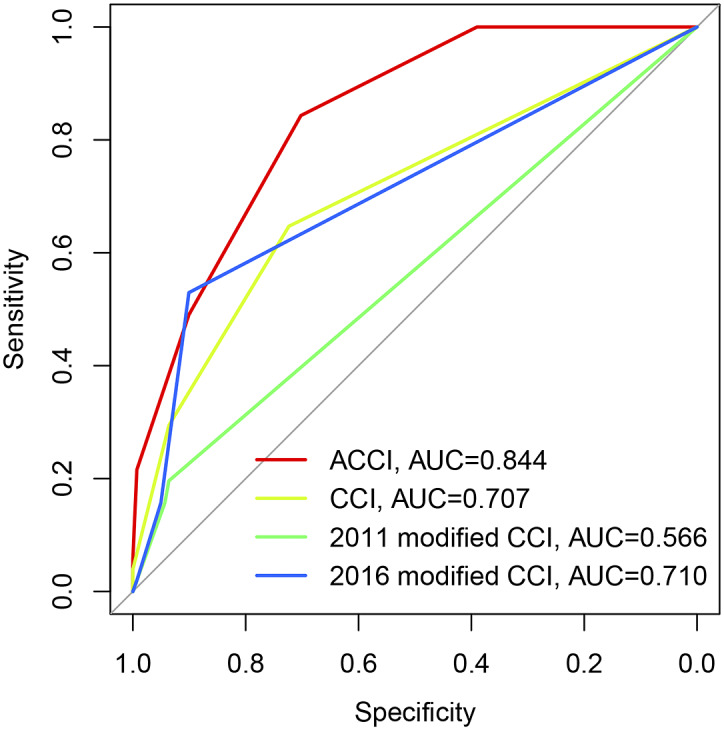
Receiver operating characteristic (ROC) curves of distinct Charlson comorbidity scores for distinguishing survivors and non-survivors of SFTS; SFTS, Severe fever with thrombocytopenia syndrome.

To explore the discriminative value of different CCI index scores in predicting poor prognosis in SFTS patients, we evaluated the differences and area under the ROC curve (AUC) of four different CCI indices in survivors and non-survivors. The results showed that the four CCI scores were significantly higher in the non-survivor group than in the survivor group (Table 3), but the highest AUC was found for the ACCI, with an AUC (95% CI) of 0.844 (0.787–0.900), indicating a higher diagnostic value. The diagnostic value of the 2011 revised CCI was poor, with an AUC (95% CI) of 0.566 (0.470–0.662). The diagnostic values of the original 1987 CCI and the 2016 revised CCI were similar, with AUCs (95% CI) of 0.707 (0.618–0.795) and 0.710 (0.619–0.800), respectively (Figure 1).

**Table 3.** Distinct Charlson comorbidity scores for survivors and non-survivors of SFTS

	Survivors (n = 141)	Non-survivors (n = 51)	*p* value
1987 original Charlson comorbidity index	0 (0, 1)	1 (0, 2)	<0.001
1994 age-adjusted Charlson comorbidity index	2 (1, 3)	3 (3, 4)	<0.001
2011 Charlson comorbidity index modified by Quan et al. [20]	0 (0, 0)	0 (0, 0)	0.011
2016 Charlson comorbidity index modified by Bannay et al. [21]	0 (0, 0)	1 (0, 1)	<0.001

### ACCI is an independent risk factor for prognosis in SFTS patients

**Figure 2.**
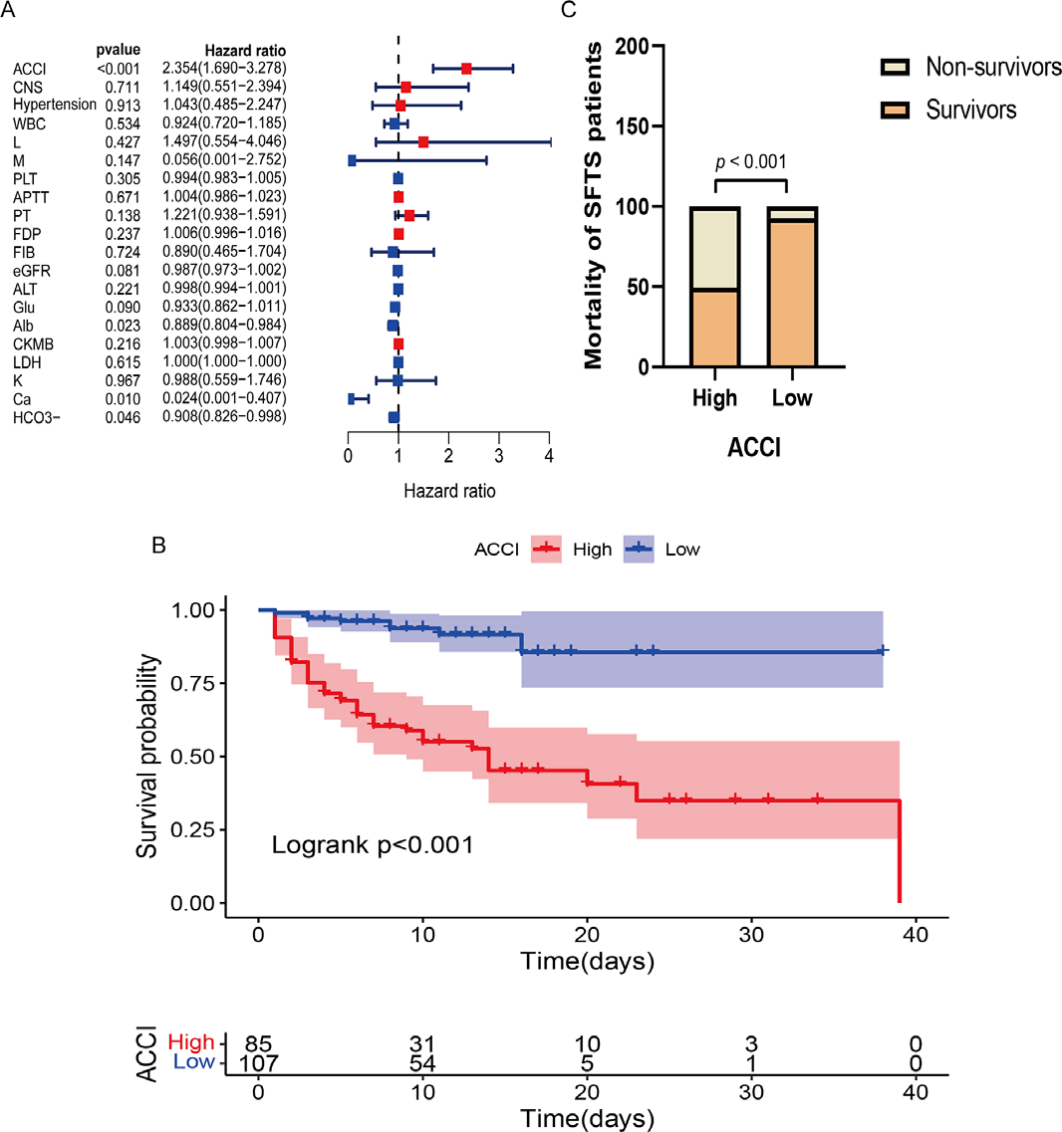
(A) Forest plots of HRs by multivariable Cox regression analysis; Red indicates HR > 1, Blue indicates HR < 1. (B) Kaplan–Meier survival curve for overall survival in SFTS patients stratified according to the ACCI. (C) Comparison of fatality at different ACCI levels.

Based on the results of the univariate analysis, variables with *p* < 0.1 were included in the multivariate Cox regression analysis. The results showed that ACCI (hazard ratio (HR) = 2.354 (95% confidence interval (CI), 1.690–3.278), *p* < 0.001) was an independent risk factor for death in SFTS patients, while Ca2+ (HR = 0.024 (95% CI, 0.001–0.407), *p* = 0.010), Alb (HR = 0.889 (95% CI, 0.804–0.984), *p* = 0.023), and HCO3- (HR = 0.908 (95% CI, 0.826–0.998), *p* = 0.046) were protective factors for SFTS mortality (Figure 2a). The optimal cutoff value for the ACCI in predicting prognosis, as identified through ROC curve analysis, was found to be 2.5, with a sensitivity of 0.843 and a specificity of 0.702 (AUC = 0.844, 95% CI = 0.787–0.900). The Kaplan–Meier survival analysis stratified by the optimal ACCI cutoff value showed that high-risk SFTS patients with ACCI >2.5 had a poor prognosis (log-rank test *p* < 0.001) (Figure 2b). Moreover, the mortality rate of SFTS patients in the high-risk ACCI group was significantly higher than that in the low-risk ACCI group (50.58% vs. 7.48%, *p* < 0.001) (Figure 2c).

## Discussion

SFTS is an emerging infectious disease with a high mortality rate, ranging from 12% to 30%. In this study, among the 192 SFTS patients included, the mortality rate was 26.6%. The lack of timely medical treatment awareness and low medical standards may contribute to the higher mortality rate of SFTS patients in this region. The clinical manifestations of SFTS are not specific, with the main symptoms including fever, nausea, and vomiting. In this study, fever was the most common symptom in SFTS patients, with a proportion as high as 95.3%. Similar to other viral infections, the digestive and nervous systems are the most common targets of the virus. In this study, gastrointestinal symptoms were the most common local symptoms and included nausea, vomiting, abdominal pain, and diarrhoea. In contrast, neurological symptoms were more common in expired SFTS patients, suggesting a poor prognosis. In addition, rash appeared in most patients. A previous study detected SFTSV RNA and virus-infected cells in skin biopsy specimens of SFTS patients [25], indicating that the SFTS virus also invades the skin during the early stage of the disease.

The main laboratory findings in SFTS patients are decreases in platelets. The current mechanism of platelet reduction is thought to be due to the SFTS virus invading and adhering to platelets, triggering macrophage recognition and phagocytosis of platelets [26]. On the other hand, SFTSV stimulation and damage to vascular endothelial cells lead to barrier function impairment and platelet adhesion [27]. Compared with SFTS survivors, the decrease in ALT and Alb in deceased patients indicated that liver function impairment was correlated with poor prognosis. Similarly, the increase in CK-MB, aPTT, PT, and FDP and the decrease in eGFR indicated that heart and kidney damage and coagulation dysfunction were related to mortality.

In addition, this study found that the blood glucose level in patients in the non-survivor group was significantly elevated, and 31.4% of patients in the non-surviving group had diabetes, which was consistent with a previous study’s conclusion that a hyperglycaemic state in STFS patients was significantly correlated with mortality. Their study suggested that elevated fasting blood glucose was related to immunity and infection, and the severity of infection led to stress-induced hyperglycaemia. Furthermore, pancreatic injuries might also lead to insulin level disruptions, resulting in hyperglycaemia [28]. Past research has shown that acute pancreatitis can be a complication of SFTS. Patients with SFTS have been found to exhibit mild pancreatic congestion and increased levels of pancreatic amylase. Additionally, real-time RT–PCR tests have confirmed the presence of SFTSV in the pancreas of these patients [29]. Hu et al. reported that 10 out of 33 SFTS patients (30.3%) had concurrent acute pancreatitis. Research by Zhang Yin et al. found elevated amylase in 59 out of 77 SFTS patients, with some also showing elevated blood sugar levels [28, 30]. The pathogenesis of SFTSV complicating acute pancreatitis is still not fully understood. Apart from the cytopathic effects of SFTSV, cytokine storms are also believed to play a crucial role in the onset of acute pancreatitis [29].

The bicarbonate levels in critically ill patients were increased. Elevated serum bicarbonate levels in hospitalized patients may be closely related to metabolic alkalosis [31]. Gastrointestinal symptoms were the main clinical manifestations in this study, which may lead to metabolic alkalosis. In this study, high serum calcium level was a protective factor for death in SFTS patients. Serum calcium affects many physiological functions of the body, including the release of hormones and neurotransmitters, activation of enzymes, and blood coagulation. The normal maintenance of serum calcium levels helps protect the body’s balance [32]. Albumin is primarily synthesized in the liver, aiding in the transport of various substances and serving as an indicator of the body’s nutritional status [33]. Hypoalbuminaemia is an indicator of liver dysfunction, malnutrition, and systemic inflammation. In this study, Alb levels were found to be lower in SFTS patients with poor clinical outcomes than in survivors. This may be related to the severe infections and multiorgan dysfunction caused by SFTSV infection [34].

In our multivariate regression analysis, we found that ACCI is an independent risk factor for mortality in patients with SFTS. Patients with an ACCI greater than 2.5 had a worse prognosis and a higher mortality rate. To our knowledge, this is the first study to evaluate comorbidities in combination with age as predictive factors for in-hospital mortality in SFTS patients.

Our research assessed the effectiveness of four distinct CCI scores in forecasting mortality among SFTS patients. When focussing on in-hospital mortality, ACCI emerged as the most accurate predictor. This suggests a link between age and mortality in SFTS cases. While older age often aligns with more comorbidities, it is important to note that many healthy elderly patients can still respond positively to comprehensive treatments and have good outcomes, in contrast to less healthy younger individuals [35]. In terms of comorbidities, we found a higher proportion of cerebrovascular and peripheral vascular diseases in deceased SFTS patients. In addition, we also found that ACCI >2.5 was observed in 42 patients (76.3%) in the non-survival group, including 16 patients with cerebrovascular disease and 5 patients with peripheral vascular disease, while an ACCI >2.5 was observed in 44 patients (30.7%) in the survival group, including 7 patients with cerebrovascular disease and 2 patients with peripheral vascular disease. In the non-survival group, SFTS patients with an ACCI >2.5 had more complications of cerebrovascular disease and peripheral vascular disease. This finding suggests that when considering the poor prognosis in SFTS, there is a need for close attention to patients with these comorbidities, and the possibility of incorporating treatment for existing comorbidities should be considered in the therapeutic regimen. Early improvement in the status of comorbidities might shift SFTS patients from having a high to low risk, thereby reducing mortality. ACCI, as a composite indicator of age and comorbidities, provides a better assessment of prognosis in SFTS patients. It may become a simple, rough, and comprehensive risk assessment method for clinicians managing SFTS patients [36].

This study has certain limitations. First, our retrospective assessment of comorbidities may underestimate their true prevalence and result in biased outcomes. Second, this is a single-centre study with a relatively small sample size. The lack of external data validation is also a limitation of this study [35]. Despite these limitations, the advantage of the ACCI score is that it does not require expensive lab tests and can be calculated through online applications and patient records. Therefore, we believe that the ACCI score might aid clinicians in assessing the severity of patients with SFTS in the context of limited resources.

## Conclusion

In summary, this research indicates that ACCI is a valuable predictor of mortality for hospitalized SFTS patients. The relationship between ACCI and the mortality rate of SFTS deserves validation in a broader population.

## Supporting information

Gong et al. supplementary material Gong et al. supplementary material

## Data Availability

The datasets used and analyzed during the current study are available from the corresponding author upon reasonable request.

## Supplementary material

For supplementary material accompanying this paper visit http://doi.org/10.1017/S0950268823001930. click here to view supplementary material
