# Supplementary material for: ACCI could be a poor prognostic indicator for the in-hospital mortality of patients with SFTS
Source: Epidemiol Infect. 2023 Dec 6;151:e203. doi: 10.1017/S0950268823001930 (PMC10753457; doi:10.1017/S0950268823001930)
Supplement: Gong et al. supplementary material [file S0950268823001930sup001.docx]

Charlson Comorbidity Index (CCI) is a standardized method for assessing comorbidities, which was originally developed to predict all-cause mortality within one year based on 17 underlying conditions [1-2]. Among them, peripheral vascular diseases mainly include arterial system diseases and venous system diseases.The arterial diseases mainly include atherosclerosis, occlusive arteriosclerosis, lower extremity arterial thrombosis, aortic aneurysm, etc. Venous system diseases are mainly varicose veins, phlebitis, and lower extremity venous thrombosis [3].

CCI was proposed in 1987 and has been validated in a number of clinical Settings. This is the most widely used comorbidity index [1-2]. Due to advances in treatment and disease management, updated versions of CCI have been adapted, validated, and reported in different databases [4-6]. We summarized the four methods of CCI score calculation based on the literature. (Supplementary Table 1)

**Supplementary Table 1.** Calculation of the four Charlson comorbidity scores

| Comorbidity | 1987  original  Charlson  index | 1994  age-  adjusted  Charlson  index | 2011  Charlson  index  modified  by Quan  et al. | 2016  Charlson  index  modified  by  Bannay  et al |
| --- | --- | --- | --- | --- |
| Myocardial infarction | 1 | 1 | 0 | 0 |
| Chronic cardiac failure | 1 | 1 | 2 | 2 |
| Peripheral vascular disease | 1 | 1 | 0 | 1 |
| Cerebrovascular disease | 1 | 1 | 0 | 1 |
| Dementia | 1 | 1 | 2 | 2 |
| Chronic pulmonary disease | 1 | 1 | 1 | 1 |
| Connective tissue disease | 1 | 1 | 1 | 0 |
| Peptic ulcer | 1 | 1 | 0 | 0 |
| Mild liver disease | 1 | 1 | 2 | 2 |
| Diabetes mellitus without chronic complications | 1 | 1 | 0 | 0 |
| Hemiplegia | 2 | 2 | 2 | 2 |
| Diabetes with end organ  damage | 2 | 2 | 1 | 0 |
| Moderate/severe renal  disease | 2 | 2 | 1 | 1 |
| tumour/Leukaemia/Lymphoma | 2 | 2 | 2 | 2 |
| Moderate/severe liver  disease | 3 | 3 | 4 | 3 |
| Metastatic solid tumour | 6 | 6 | 6 | 11 |
| AIDS | 6 | 6 | 4 | 1 |
| Age |  |  |  |  |
| <50 years | - | 0 | - | - |
| 50–59 years | - | 1 | - | - |
| 60–69 years | - | 2 | - | - |
| 70–79 years | - | 3 | - | - |
| ≥80 years | - | 4 | - | - |

References

[1] Setter NW, et al. (2020) Charlson comorbidity index scores and in-hospital prognosis of patients with severe acute respiratory infections. *Internal Medicine Journal* 50(6), 691-7.

[2] Charlson ME, et al. (1987) A new method of classifying prognostic comorbidity in longitudinal studies: development and validation. *Journal of Chronic Diseases* 40, 373–83.

[3] Swenty CF and Hall M. (2020) Peripheral Vascular Disease. *Home Healthc Now* 38(6), 294-301

[4] Charlson M, et al. (1994) Validation of a combined comorbidity index. *Journal of Clinical Epidemiology* 47, 1245–51.

[5] Quan H, et al. (2011) Updating and validating the Charlson comorbidity index and score for risk adjustment in hospital discharge abstracts using data from 6 countries. *American Journal of Epidemiology* 173(6), 676-82.

[6] Bannay A, et al. (2016) The best use of the Charlson comorbidity index with electronic health care database to predict mortality. *Medical Care* 54, 188–94.
